# Successful Treatment of Disseminated Fusariosis with the Combination of Voriconazole and Liposomal Amphotericin B

**DOI:** 10.4274/tjh.2016.0128

**Published:** 2016-12-01

**Authors:** Nur Efe İris, Serkan Güvenç, Tülay Özçelik, Aslıhan Demirel, Safiye Koçulu, Esin Çevik, Mutlu Arat

**Affiliations:** 1 İstanbul Bilim University Faculty of Medicine, Department of Infectious Diseases and Clinical Microbiology, İstanbul, Turkey; 2 İstanbul Bilim University Faculty of Medicine, Department of Hematology, İstanbul, Turkey

**Keywords:** invasive fungal infection, Fusariosis, Combined antifungal treatment, Lyposomal amphotericin B, Voriconazole, Acute myeloid leukemia

## To the Editor,

Fusarium species are important causes of disseminated infections in patients with prolonged, severe neutropenia. Clinical presentation includes refractory fever, skin lesions, and sinopulmonary infections [1,2]. Disseminated Fusarium infection (DFI) carries a poor prognosis, which is related to the angiotropism of Fusarium and its capacity for adventitious sporulation in tissues [3] and resistance to many antifungal agents [4].

Here we report a hematopoietic stem cell transplant (HSCT) recipient with acute myeloid leukemia (AML) and disseminated fusariosis who was successfully treated using both liposomal amphotericin B and voriconazole.

A 24-year-old male patient underwent allogeneic HSCT from his HLA-matched brother for AML in the first remission. At 21 months after HSCT he had extramedullary relapse with a mass over his humerus. He received radiotherapy plus the FLAG-IDA salvage regimen. After 4 months, medullary relapse occurred.

**Figure 1:**
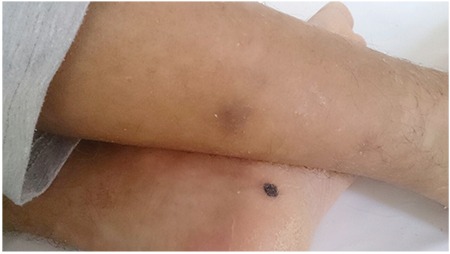
Eschar formation on the foot and papules over the leg.

When he was hospitalized for the medullary relapse, he received clofarabine with ARA-C, which caused severe neutropenia and fever. According to in-house protocol for neutropenia, piperacillin-tazobactam was initiated. However, on the third day, he was still febrile and neutropenic, so treatment was changed to meropenem and 2 days later amikacin was added. Because of hypotension, we broadened the spectrum with vancomycin. He was still febrile and he had rectal carbapenem-resistant Klebsiella pneumoniae colonization. Antibiotherapy was reordered with colistin plus meropenem and vancomycin. According to thorax computed tomography findings that showed a nodule on the base of the left lung and sphenoidal sinusitis, 3 mg/kg liposomal amphotericin B was added empirically to his treatment. On follow-up, new papular and nodular skin lesions appeared on his face, head, arms, legs, feet, and anterior-posterior trunk. Some of these papules had central necrosis and eschar formations on his feet (Figure 1). These papules and especially the nodules were extremely painful, and he also had myalgia. Blood cultures revealed Fusarium solani by the VITEK system and MALDI-TOF. The diagnosis of DFI was established and we decided to augment the antifungal therapy on the seventh day by adding intravenous voriconazole as Fusarium is a resistant pathogen and the prognosis is especially poor in neutropenic patients. There were no antifungal susceptibility test results for amphotericin B or voriconazole. The skin lesions were not biopsied or cultured. Five days later his skin lesions began to resolve and on the sixth day of combined antifungal therapy his fever subsided. He was neutropenic at the time and neutrophil levels resolved 5 days later when he was afebrile. Clinical improvement was evident 5 days before the resolution of neutropenia. Parenteral antifungal treatment was continued for 21 days and the patient was discharged on oral voriconazole treatment. After combined antifungal therapy, blood cultures obtained on the fifth day were negative.

We added voriconazole to the antifungal treatment of this patient because disseminated fusariosis has a very poor prognosis. Some investigators have stated that antifungal therapy is rarely effective and recovery depends on neutrophil recovery, but we achieved effective control of fusariosis with combined antifungal therapy before neutrophil recovery [5,6,7,8,9,10].

In conclusion, using combination therapy such as amphotericin B and voriconazole may be considered as early as possible in patients who are not responding to antifungal monotherapy.
